# Supplementary material for: A set of multi-entry identification keys to African frugivorous flies (Diptera, Tephritidae)
Source: Zookeys. 2014 Jul 24;(428):97–108. doi: 10.3897/zookeys.428.7366 (PMC4143993; doi:10.3897/zookeys.428.7366)
Supplement: Supplementary material 4 — Key to Capparimyia [file zookeys-428-097-s004.zip › SF4_ZooKeys_key to Capparimyia/lucid_player/help/help/whskin_banner.htm]

About WebHelp                                                                                   


|  |  |  |
| --- | --- | --- |
|  |  |  |
|  |  |  |
|  | Centre for Biological Information Technology  Copyright � 2004  Author: Centre for Biological Information Technology |  |
|  | Powered by: WebHelp 5.50  Generated by: RoboHelp X5  www.ehelp.com |  |
